# Immune status at pre-treatment impacts on progression-free survival of metastatic colorectal cancer patients treated with first-line chemotherapy

**DOI:** 10.1186/2051-1426-3-S2-P108

**Published:** 2015-11-04

**Authors:** Kohei Tada, Shigehisa Kitano, Hirokazu Shoji, Takashi Nishimura, Yasuhiro Shimada, Kengo Nagashima, Yoshitaka Honma, Satoru Iwasa, Natsuko Okita, Atsuo Takashima, Ken Kato, Yasuhide Yamada, Naoyuki Katayama, Narikazu Boku, Tetsuya Hamaguchi, Yuji Heike

**Affiliations:** 1 St. Lukes's International Hosp., Immunotherapy & Cell Therapy, Tokyo, Japan; 2 Dept. of Exp. Therap., Natl. Cancer Ctr. Hosp., Tokyo, Japan; 3 National Cancer Center Hospital, Tokyo, Japan; 4 Tokyo Jikei Medical University, Tokyo, Japan; 5 Chiba University, Chiba, Japan; 6 Mie University Graduate School of Medicine, Tsu, Japan

## Background

Immunological status in surgically resected specimens affects the outcome of ‘operable’ colorectal cancer patients. For example, a high number of tumor infiltrating lymphocytes, CD3^+^ cells, CD8^+^ cells, or CD45RO^+^ cells in a tumor is associated with improved prognosis. However, it has not been determined whether the immunological status in peripheral blood affects the outcome of ‘inoperable’ metastatic colorectal cancer (MCRC) patients. We investigated the impact of peripheral immunological status at pre-treatment on progression-free survival (PFS) of MCRC patients.

## Methods

Peripheral blood was prospectively collected from consecutive MCRC patients (n=40) before they received a first-line chemotherapy. The quantity of each of 25 immune subsets, including monocytic myeloid-derived suppressor cells (M-MDSC, defined as Lineage^-^CD14^+^CD11b^+^CD33^+^HLA-DR^low/-^) and effector memory T cells (T_EM_, defined as CD3^+^CD4^+^ or CD8^+^CD45RA^-^CCR7^-^) was measured using multicolor-flow cytometry. The patients were divided into high (> median) and low (< median) groups based on the median value for each immune subset. PFS was compared between the two patient groups.

## Results

Of the 25 immune subsets quantified, we identified a high quantity of M-MDSC, a low quantity of CD4^+^ T_EM_, or a low quantity of CD8^+^ T_EM_ as adverse prognostic factors for PFS. Thus, patients with high M-MDSC, low CD4^+^ T_EM_ or low CD8^+^ T_EM_ had significantly shorter PFS than those with low M-MDSC, high CD4^+^ T_EM_, or high CD8^+^ T_EM_, respectively (p=0.004, 0.005, and 0.002, respectively). Ten (25%) patients had three adverse factors, 11 (27.5 %) patients had 2, 8 (20%) patients had one, and 11 (27.5%) patients had none. Patients were classified into two distinct prognostic groups based on the number of adverse factors that were present in each patient. The presence of 2 or 3 adverse factors (n=21, 52.5%) correlated with significantly shorter PFS compared to the presence of no or 1 adverse factor (n=19, 47.5%) (p=0.00001). In addition, multivariate analysis showed that the presence of 2 or 3 adverse factors was an independent poor prognostic factor for PFS (Hazard ratio, 10.2; 95% confidence interval, 2.9-35.9; p=0.0003) after adjustment for previously known prognostic factors.

## Conclusions

These results suggest that peripheral immune status at pre-treatment impacts on the prognosis of MCRC patients treated with first-line chemotherapy.

